# Supplementary material for: House Dust Avoidance during Pregnancy and Subsequent Infant Development: The Japan Environment and Children’s Study
Source: Int J Environ Res Public Health. 2021 Apr 17;18(8):4277. doi: 10.3390/ijerph18084277 (PMC8073752; doi:10.3390/ijerph18084277)
Supplement: Supplementary file 1 [file ijerph-18-04277-s001.zip › ijerph-1157034-SI.pdf]

Table S1. Characteristics of the participants with respect to frequency of vacuum cleaning of the living room.

Variable	Category	Frequency of cleaning the floor of the living room with a vacuum cleaner									Cramer's
		≤ 1–2 times / month		Once a week		Several times / week		Everyday			
		N	(%)	N	(%)	N	(%)	N	(%)	*p*	*V*
Subtotal		6,795	(8.4)	25,367	(31.3)	35,362	(43.6)	13,582	(16.7)		
Age, y	<25	947	(13.9)	2,192	(8.6)	3,515	(9.9)	1,317	(9.7)	< 0.001	0.04
	25–<30	2,219	(32.7)	7,482	(29.5)	9,878	(27.9)	3,490	(25.7)		
	30–<35	2,228	(32.8)	9,125	(36.0)	12,687	(35.9)	4,847	(35.7)		
	≥35	1,401	(20.6)	6,567	(25.9)	9,277	(26.2)	3,926	(28.9)		
Body mass index, kg/m^2	<18.5	1,058	(15.6)	4,004	(15.8)	5,672	(16.1)	2,395	(17.7)	< 0.001	0.02
	18.5 – <25	4,887	(72.0)	18,959	(74.8)	25,998	(73.6)	9,878	(72.8)		
	≥25	846	(12.5)	2,394	(9.4)	3,675	(10.4)	1,297	(9.6)		
Parity	Primipara	4,390	(67.3)	13,814	(56.3)	13,273	(38.4)	2,773	(20.7)	< 0.001	0.28
	Multipara	2,137	(32.7)	10,734	(43.7)	21,311	(61.6)	10,642	(79.3)		
History of allergy	No	3,466	(51.3)	12,577	(49.8)	17,427	(49.5)	6,755	(49.9)	0.058	0.01
	Yes	3,293	(48.7)	12,682	(50.2)	17,796	(50.5)	6,777	(50.1)		
Number of hours spent outdoors, hour	<5	4,464	(65.8)	18,380	(72.5)	25,406	(71.9)	9,839	(72.5)	< 0.001	0.03
	5–12	1,997	(29.5)	6,331	(25.0)	8,841	(25.0)	3,313	(24.4)		
	≥13	321	(4.7)	638	(2.5)	1,073	(3.0)	417	(3.1)		
Smoking status	Never	4,191	(62.1)	15,966	(63.3)	20,065	(57.2)	7,172	(53.3)	< 0.001	0.05
	Former	2,257	(33.4)	8,484	(33.6)	13,489	(38.5)	5,647	(42.0)		
	Current	306	(4.5)	788	(3.1)	1,508	(4.3)	643	(4.8)		
Alcohol intake	Never	2,185	(32.4)	8,227	(32.7)	11,707	(33.4)	4,796	(35.6)	< 0.001	0.03
	Former	4,406	(65.3)	16,403	(65.1)	22,368	(63.7)	8,210	(61.0)		
	Current	154	(2.3)	562	(2.2)	1,031	(2.9)	465	(3.5)		
Number of hours spent outdoors	<1	1,657	(25.4)	6,046	(24.8)	5,490	(16.1)	1,712	(13.1)	< 0.001	0.09
	1–<2	3,078	(47.1)	11,885	(48.8)	16,494	(48.4)	6,054	(46.2)		
	2–<3	822	(12.6)	2,924	(12.0)	5,845	(17.2)	2,715	(20.7)		
	≥3	980	(15.0)	3,514	(14.4)	6,226	(18.3)	2,618	(20.0)		
Physical activity	No	1,857	(28.1)	6,668	(27.1)	6,974	(20.4)	2,542	(19.4)	< 0.001	0.08
	Yes	4,748	(71.9)	17,959	(72.9)	27,244	(79.6)	10,584	(80.6)		
Quintile of folic acid intake, µg	≤153	1,873	(27.6)	5,352	(21.1)	6,449	(18.2)	2,090	(15.4)	< 0.001	0.06
	154–203	1,477	(21.7)	5,516	(21.8)	6,826	(19.3)	2,466	(18.2)		
	204–257	1,318	(19.4)	5,179	(20.4)	7,267	(20.6)	2,636	(19.4)		
	258–337	1,123	(16.5)	4,982	(19.6)	7,400	(20.9)	2,920	(21.5)		
	≥338	1,003	(14.8)	4,336	(17.1)	7,418	(21.0)	3,467	(25.5)		
Marital status	Married	6,250	(93.0)	24,196	(96.1)	33,608	(95.8)	12,970	(96.3)	< 0.001	0.04
	Single	418	(6.2)	851	(3.4)	1,169	(3.3)	365	(2.7)		
	Divorced or widowed	51	(0.8)	133	(0.5)	304	(0.9)	140	(1.0)		
Highest education level, y	≤12	2,446	(36.1)	7,637	(30.2)	12,740	(36.2)	5,528	(40.9)	< 0.001	0.07
	>12 – <16	2,739	(40.4)	10,806	(42.7)	15,326	(43.5)	5,542	(41.0)		
	≥16	1,588	(23.5)	6,859	(27.1)	7,167	(20.3)	2,453	(18.1)		
Employed	No	2,153	(31.9)	7,030	(27.9)	18,258	(52.0)	9,217	(68.4)	< 0.001	0.30
	Yes	4,592	(68.1)	18,190	(72.1)	16,833	(48.0)	4,263	(31.6)		
Annual household income, million yen	<4	2,616	(41.7)	8,143	(34.1)	13,805	(41.8)	5,339	(42.5)	< 0.001	0.08
	4 – <6	1,929	(30.8)	7,711	(32.3)	11,180	(33.9)	4,403	(35.0)		
	≥6	1,725	(27.5)	8,036	(33.6)	8,031	(24.3)	2,836	(22.6)		
Type of residence	Wooden detached house	2,574	(38.1)	10,057	(39.8)	14,727	(41.9)	5,922	(43.8)	< 0.001	0.03
	Steel-frame detached house	343	(5.1)	1,569	(6.2)	2,269	(6.5)	927	(6.9)		
	Wooden multiple dwelling house/apartment	989	(14.7)	3,392	(13.4)	4,206	(12.0)	1,357	(10.0)		
	Steel-frame multiple dwelling house/apartment	2,761	(40.9)	10,038	(39.7)	13,626	(38.7)	5,180	(38.3)		
	Other	84	(1.2)	206	(0.8)	351	(1.0)	130	(1.0)		
Number of rooms in the house/apartment	≤2	1,830	(27.1)	5,187	(20.5)	6,214	(17.7)	1,981	(14.7)	< 0.001	0.06
	3	2,098	(31.0)	8,506	(33.7)	11,319	(32.2)	4,228	(31.3)		
	4	979	(14.5)	4,449	(17.6)	6,978	(19.8)	2,983	(22.1)		
	5	802	(11.9)	3,485	(13.8)	5,551	(15.8)	2,255	(16.7)		
	≥6	1,053	(15.6)	3,648	(14.4)	5,110	(14.5)	2,072	(15.3)		
Living room flooring materials	Tatami (Japanese straw floor covering)	909	(13.4)	2,829	(11.2)	3,892	(11.0)	1,531	(11.3)	< 0.001	0.03
	Carpet on tatami	746	(11.0)	2,236	(8.8)	3,081	(8.7)	1,123	(8.3)		
	Wooden flooring / tiles	2,122	(31.3)	8,638	(34.1)	12,626	(35.8)	5,317	(39.2)		
	Carpet on wooden flooring/tiles	2,885	(42.6)	11,201	(44.2)	15,025	(42.6)	5,279	(39.0)		
	Other	118	(1.7)	414	(1.6)	634	(1.8)	301	(2.2)		
Having a pet	No	5,166	(76.2)	20,230	(79.9)	27,198	(77.1)	9,909	(73.1)	< 0.001	0.05
	Yes	1,616	(23.8)	5,104	(20.2)	8,090	(22.9)	3,641	(26.9)		
Usage of air purifiers	Yes	2,815	(41.5)	12,216	(48.2)	18,548	(52.5)	7,573	(55.8)	< 0.001	0.08
	No	3,973	(58.5)	13,112	(51.8)	16,756	(47.5)	5,989	(44.2)		
Age of house/apartment building, y	<1	293	(4.3)	1,418	(5.6)	2,127	(6.1)	755	(5.6)	< 0.001	0.04
	1 – <3	581	(8.6)	2,867	(11.4)	4,132	(11.8)	1,558	(11.5)		
	3 – <5	478	(7.1)	2,355	(9.3)	3,440	(9.8)	1,428	(10.6)		
	5– <10	950	(14.1)	4,025	(16.0)	5,318	(15.1)	2,226	(16.5)		
	10 – <20	1,581	(23.4)	6,003	(23.8)	8,206	(23.3)	3,039	(22.5)		
	≥20	2,052	(30.4)	6,300	(25.0)	8,606	(24.5)	3,295	(24.4)		
	Unknown	810	(12.0)	2,258	(9.0)	3,342	(9.5)	1,227	(9.1)		
House renovation/interior finishing after getting pregnant	Yes	215	(3.2)	760	(3.0)	1,136	(3.2)	449	(3.3)	0.312	0.01
	No	6,548	(96.8)	24,493	(97.0)	34,044	(96.8)	13,056	(96.7)		
Number of years living in the current place of residence	<1	488	(7.4)	1,734	(7.0)	2,415	(7.1)	736	(5.6)	< 0.001	0.05
	1 – <3	3,046	(46.1)	11,367	(46.0)	14,641	(42.8)	5,018	(38.0)		
	3 – <5	1,303	(19.7)	5,408	(21.9)	7,867	(23.0)	3,262	(24.7)		
	5– <10	978	(14.8)	4,011	(16.2)	6,172	(18.0)	2,857	(21.6)		
	10 – <20	383	(5.8)	1,175	(4.8)	1,833	(5.4)	811	(6.1)		
	≥20	413	(6.3)	1,014	(4.1)	1,303	(3.8)	516	(3.9)		

Table S2. Characteristics of the participants with respect to frequency of vacuum cleaning of the bedding

Variable	Category	Frequency of cleaning the futon with a vacuum cleaner									
		≤ 1–2 times / month		Once a week		Several times / week		Everyday			Cramer's
		N	(%)	N	(%)	N	(%)	N	(%)	*p*	*V*
Subtotal		46,045	(56.8)	10,286	(12.7)	13,265	(16.4)	11,510	(14.2)		
Age, y	<25	3,958	(8.6)	790	(7.7)	1,495	(11.3)	1,728	(15.0)	< 0.001	0.05
	25–<30	13,067	(28.4)	2,701	(26.3)	3,825	(28.8)	3,476	(30.2)		
	30–<35	16,642	(36.2)	3,765	(36.6)	4,703	(35.5)	3,777	(32.8)		
	≥35	12,372	(26.9)	3,030	(29.5)	3,240	(24.4)	2,529	(22.0)		
Body mass index, kg/m^2	<18.5	7,449	(16.2)	1,617	(15.7)	2,098	(15.8)	1,965	(17.1)	< 0.001	0.02
	18.5 – <25	34,205	(74.3)	7,565	(73.6)	9,697	(73.2)	8,255	(71.8)		
	≥25	4,367	(9.5)	1,101	(10.7)	1,462	(11.0)	1,282	(11.2)		
Parity	Primipara	20,700	(46.3)	4,116	(40.9)	5,230	(40.3)	4,204	(37.2)	< 0.001	0.07
	Multipara	24,058	(53.8)	5,939	(59.1)	7,739	(59.7)	7,088	(62.8)		
History of allergy	No	23,307	(50.8)	4,856	(47.4)	6,413	(48.5)	5,649	(49.3)	< 0.001	0.03
	Yes	22,546	(49.2)	5,391	(52.6)	6,801	(51.5)	5,810	(50.7)		
Number of hours spent outdoors, hour	<5	33,330	(72.5)	7,499	(73.0)	9,354	(70.6)	7,906	(68.8)	< 0.001	0.02
	5–12	11,313	(24.6)	2,527	(24.6)	3,504	(26.5)	3,138	(27.3)		
	≥13	1,356	(3.0)	253	(2.5)	392	(3.0)	448	(3.9)		
Smoking status	Never	28,020	(61.3)	6,192	(60.6)	7,399	(56.1)	5,783	(50.8)	< 0.001	0.06
	Former	16,162	(35.3)	3,650	(35.7)	5,178	(39.3)	4,887	(43.0)		
	Current	1,557	(3.4)	373	(3.7)	608	(4.6)	707	(6.2)		
Alcohol intake	Never	15,266	(33.4)	3,400	(33.2)	4,391	(33.4)	3,858	(33.9)	0.841	0.00
	Former	29,184	(63.8)	6,542	(64.0)	8,420	(63.9)	7,241	(63.6)		
	Current	1,275	(2.8)	287	(2.8)	357	(2.7)	293	(2.6)		
Number of hours spent outdoors	<1	9,228	(20.8)	1,985	(20.0)	2,121	(16.6)	1,571	(14.2)	< 0.001	0.05
	1–<2	21,284	(48.0)	5,002	(50.5)	6,181	(48.4)	5,044	(45.6)		
	2–<3	6,639	(15.0)	1,464	(14.8)	2,169	(17.0)	2,034	(18.4)		
	≥3	7,166	(16.2)	1,461	(14.7)	2,301	(18.0)	2,410	(21.8)		
Physical activity	No	10,981	(24.6)	2,255	(22.7)	2,705	(21.1)	2,100	(18.9)	< 0.001	0.05
	Yes	33,686	(75.4)	7,697	(77.3)	10,140	(78.9)	9,012	(81.1)		
Quintile of folic acid intake, µg	≤153	9,250	(20.1)	1,923	(18.7)	2,455	(18.5)	2,136	(18.6)	< 0.001	0.03
	154–203	9,430	(20.5)	2,053	(20.0)	2,658	(20.0)	2,144	(18.6)		
	204–257	9,364	(20.3)	2,167	(21.1)	2,700	(20.4)	2,169	(18.9)		
	258–337	9,213	(20.0)	2,106	(20.5)	2,763	(20.8)	2,343	(20.4)		
	≥338	8,783	(19.1)	2,037	(19.8)	2,687	(20.3)	2,717	(23.6)		
Marital status	Married	43,729	(95.8)	9,883	(96.8)	12,625	(95.9)	10,787	(94.6)	< 0.001	0.02
	Single	1,605	(3.5)	272	(2.7)	446	(3.4)	480	(4.2)		
	Divorced or widowed	330	(0.7)	59	(0.6)	97	(0.7)	142	(1.2)		
Highest education level, y	≤12	14,942	(32.6)	3,408	(33.2)	4,953	(37.5)	5,048	(44.0)	< 0.001	0.07
	>12 – <16	19,827	(43.2)	4,519	(44.1)	5,498	(41.6)	4,569	(39.9)		
	≥16	11,129	(24.3)	2,326	(22.7)	2,766	(20.9)	1,846	(16.1)		
Employed	No	20,004	(43.7)	4,467	(43.6)	6,210	(47.2)	5,977	(52.5)	< 0.001	0.06
	Yes	25,732	(56.3)	5,771	(56.4)	6,962	(52.9)	5,413	(47.5)		
Annual household income, million yen	<4	16,385	(38.0)	3,480	(36.1)	5,047	(40.7)	4,991	(47.1)	< 0.001	0.05
	4 – <6	14,393	(33.4)	3,310	(34.3)	4,177	(33.7)	3,343	(31.6)		
	≥6	12,344	(28.6)	2,854	(29.6)	3,173	(25.6)	2,257	(21.3)		
Type of residence	Wooden detached house	18,963	(41.4)	4,542	(44.3)	5,429	(41.1)	4,346	(38.0)	< 0.001	0.02
	Steel-frame detached house	2,911	(6.4)	626	(6.1)	852	(6.5)	719	(6.3)		
	Wooden multiple dwelling house/apartment	5,450	(11.9)	1,239	(12.1)	1,694	(12.8)	1,561	(13.7)		
	Steel-frame multiple dwelling house/apartment	18,032	(39.4)	3,752	(36.6)	5,122	(38.8)	4,699	(41.1)		
	Other	448	(1.0)	96	(0.9)	122	(0.9)	105	(0.9)		
Number of rooms in the house/apartment	≤2	8,699	(19.0)	1,725	(16.8)	2,452	(18.6)	2,336	(20.4)	< 0.001	0.02
	3	14,828	(32.4)	3,191	(31.1)	4,269	(32.3)	3,863	(33.8)		
	4	8,712	(19.0)	1,986	(19.4)	2,547	(19.3)	2,144	(18.7)		
	5	6,822	(14.9)	1,687	(16.5)	2,049	(15.5)	1,535	(13.4)		
	≥6	6,767	(14.8)	1,657	(16.2)	1,895	(14.3)	1,564	(13.7)		
Living room flooring materials	Tatami (Japanese straw floor covering)	5,152	(11.2)	1,120	(10.9)	1,479	(11.2)	1,410	(12.3)	< 0.001	0.01
	Carpet on tatami	4,029	(8.8)	842	(8.2)	1,203	(9.1)	1,112	(9.7)		
	Wooden flooring / tiles	16,385	(35.7)	3,621	(35.3)	4,642	(35.1)	4,055	(35.3)		
	Carpet on wooden flooring/tiles	19,542	(42.5)	4,456	(43.5)	5,682	(42.9)	4,710	(41.0)		
	Other	830	(1.8)	217	(2.1)	227	(1.7)	193	(1.7)		
Having a pet	No	35,802	(77.9)	7,907	(77.0)	10,145	(76.6)	8,649	(75.4)	< 0.001	0.02
	Yes	10,169	(22.1)	2,356	(23.0)	3,096	(23.4)	2,830	(24.7)		
Usage of air purifiers	Yes	21,764	(47.3)	5,466	(53.2)	7,373	(55.7)	6,549	(57.0)	< 0.001	0.08
	No	24,220	(52.7)	4,805	(46.8)	5,868	(44.3)	4,937	(43.0)		
Age of house/apartment building, y	<1	2,590	(5.7)	537	(5.2)	838	(6.4)	628	(5.5)	< 0.001	0.03
	1 – <3	5,369	(11.7)	1,215	(11.9)	1,424	(10.8)	1,130	(9.9)		
	3 – <5	4,426	(9.7)	1,035	(10.1)	1,256	(9.5)	984	(8.6)		
	5– <10	7,144	(15.6)	1,666	(16.3)	1,988	(15.1)	1,721	(15.1)		
	10 – <20	10,567	(23.1)	2,424	(23.7)	3,099	(23.5)	2,739	(24.0)		
	≥20	11,526	(25.2)	2,616	(25.5)	3,305	(25.1)	2,806	(24.6)		
	Unknown	4,186	(9.1)	753	(7.4)	1,279	(9.7)	1,419	(12.4)		
House renovation/interior finishing after getting pregnant	Yes	1,396	(3.1)	329	(3.2)	442	(3.4)	393	(3.4)	0.098	0.01
	No	44,424	(97.0)	9,913	(96.8)	12,746	(96.7)	11,058	(96.6)		
Number of years living in the current place of residence	<1	3,072	(6.9)	606	(6.0)	867	(6.8)	828	(7.5)	< 0.001	0.02
	1 – <3	19,663	(43.9)	4,103	(40.7)	5,623	(43.9)	4,683	(42.2)		
	3 – <5	10,042	(22.4)	2,402	(23.8)	2,875	(22.4)	2,521	(22.7)		
	5– <10	7,797	(17.4)	1,953	(19.4)	2,251	(17.6)	2,017	(18.2)		
	10 – <20	2,311	(5.2)	589	(5.8)	690	(5.4)	612	(5.5)		
	≥20	1,879	(4.2)	429	(4.3)	513	(4.0)	425	(3.8)		

Table S3. Characteristics of the participants with respect to the frequency of futon airing

Variable	Category	Frequency of airing the futon									
		≤ 1–2 times / month		Once a week		Several times / week		Everyday			Cramer's
		N	(%)	N	(%)	N	(%)	N	(%)	*p*	*V*
Subtotal		9,591	(11.8)	15,673	(19.3)	30,118	(37.1)	25,724	(31.7)		
Age, y	<25	901	(9.4)	1,341	(8.6)	2,971	(9.9)	2,758	(10.7)	< 0.001	0.02
	25–<30	2,781	(29.0)	4,524	(28.9)	8,659	(28.8)	7,105	(27.6)		
	30–<35	3,264	(34.0)	5,641	(36.0)	10,806	(35.9)	9,176	(35.7)		
	≥35	2,645	(27.6)	4,166	(26.6)	7,679	(25.5)	6,681	(26.0)		
Body mass index, kg/m^2	<18.5	1,581	(16.5)	2,557	(16.3)	4,782	(15.9)	4,209	(16.4)	0.047	0.01
	18.5 – <25	7,090	(74.0)	11,454	(73.1)	22,214	(73.8)	18,964	(73.8)		
	≥25	912	(9.5)	1,652	(10.6)	3,108	(10.3)	2,540	(9.9)		
Parity	Primipara	5,109	(55.3)	7,690	(50.5)	13,015	(44.3)	8,436	(33.5)	< 0.001	0.15
	Multipara	4,134	(44.7)	7,554	(49.6)	16,364	(55.7)	16,772	(66.5)		
History of allergy	No	4,636	(48.6)	7,725	(49.5)	14,984	(50.0)	12,880	(50.3)	0.023	0.01
	Yes	4,912	(51.5)	7,895	(50.5)	15,016	(50.1)	12,725	(49.7)		
Number of hours spent outdoors, hour	<5	6,502	(67.9)	11,329	(72.4)	21,719	(72.2)	18,539	(72.2)	< 0.001	0.02
	5–12	2,710	(28.3)	3,884	(24.8)	7,548	(25.1)	6,340	(24.7)		
	≥13	367	(3.8)	446	(2.9)	823	(2.7)	813	(3.2)		
Smoking status	Never	5,729	(60.2)	9,483	(60.9)	17,750	(59.3)	14,432	(56.6)	< 0.001	0.03
	Former	3,428	(36.0)	5,567	(35.7)	10,986	(36.7)	9,896	(38.8)		
	Current	363	(3.8)	530	(3.4)	1,181	(4.0)	1,171	(4.6)		
Alcohol intake	Never	3,101	(32.6)	5,035	(32.3)	10,086	(33.7)	8,693	(34.1)	< 0.001	0.01
	Former	6,208	(65.2)	10,119	(64.9)	18,987	(63.5)	16,073	(63.0)		
	Current	214	(2.3)	436	(2.8)	825	(2.8)	737	(2.9)		
Number of hours spent outdoors	<1	2,370	(25.7)	3,774	(25.1)	5,455	(18.8)	3,306	(13.4)	< 0.001	0.09
	1–<2	4,373	(47.4)	7,350	(48.8)	14,346	(49.5)	11,442	(46.2)		
	2–<3	1,145	(12.4)	1,895	(12.6)	4,485	(15.5)	4,781	(19.3)		
	≥3	1,331	(14.4)	2,042	(13.6)	4,728	(16.3)	5,237	(21.2)		
Physical activity	No	2,567	(27.6)	4,003	(26.3)	6,702	(22.9)	4,769	(19.2)	< 0.001	0.07
	Yes	6,726	(72.4)	11,207	(73.7)	22,520	(77.1)	20,082	(80.8)		
Quintile of folic acid intake, μg	≤153	2,366	(24.7)	3,504	(22.4)	5,803	(19.3)	4,091	(15.9)	< 0.001	0.07
	154–203	2,114	(22.0)	3,457	(22.1)	6,131	(20.4)	4,583	(17.8)		
	204–257	1,863	(19.4)	3,245	(20.7)	6,155	(20.4)	5,137	(20.0)		
	258–337	1,719	(17.9)	2,951	(18.8)	6,214	(20.6)	5,541	(21.5)		
	≥338	1,529	(15.9)	2,514	(16.0)	5,812	(19.3)	6,369	(24.8)		
Marital status	Married	9,034	(95.1)	14,906	(95.8)	28,672	(95.9)	24,412	(95.7)	< 0.001	0.02
	Single	391	(4.1)	560	(3.6)	1,034	(3.5)	818	(3.2)		
	Divorced or widowed	77	(0.8)	94	(0.6)	188	(0.6)	269	(1.1)		
Highest education level, y	≤12	3,225	(33.7)	5,301	(33.9)	10,299	(34.3)	9,526	(37.2)	< 0.001	0.03
	>12 – <16	3,944	(41.3)	6,806	(43.5)	12,775	(42.5)	10,888	(42.5)		
	≥16	2,389	(25.0)	3,528	(22.6)	6,954	(23.2)	5,196	(20.3)		
Employed	No	3,960	(41.6)	5,912	(37.9)	12,894	(43.1)	13,892	(54.5)	< 0.001	0.13
	Yes	5,551	(58.4)	9,670	(62.1)	17,039	(56.9)	11,618	(45.5)		
Annual household income, million yen	<4	3,315	(36.9)	5,390	(36.9)	10,830	(38.4)	10,368	(43.3)	< 0.001	0.05
	4 – <6	2,900	(32.3)	4,771	(32.6)	9,525	(33.8)	8,027	(33.5)		
	≥6	2,764	(30.8)	4,455	(30.5)	7,845	(27.8)	5,564	(23.2)		
Type of residence	Wooden detached house	3,411	(35.8)	7,149	(45.8)	12,684	(42.3)	10,036	(39.2)	< 0.001	0.06
	Steel-frame detached house	498	(5.2)	877	(5.6)	1,985	(6.6)	1,748	(6.8)		
	Wooden multiple dwelling house/apartment	1,869	(19.6)	2,129	(13.7)	3,393	(11.3)	2,553	(10.0)		
	Steel-frame multiple dwelling house/apartment	3,653	(38.3)	5,292	(33.9)	11,640	(38.8)	11,020	(43.1)		
	Other	102	(1.1)	154	(1.0)	282	(0.9)	233	(0.9)		
Number of rooms in the house/apartment	≤2	2,333	(24.5)	2,959	(19.0)	5,477	(18.3)	4,443	(17.4)	< 0.001	0.05
	3	3,222	(33.8)	4,704	(30.2)	9,484	(31.6)	8,741	(34.2)		
	4	1,677	(17.6)	2,749	(17.6)	5,643	(18.8)	5,320	(20.8)		
	5	1,156	(12.1)	2,384	(15.3)	4,667	(15.6)	3,886	(15.2)		
	≥6	1,152	(12.1)	2,808	(18.0)	4,720	(15.7)	3,203	(12.5)		
Living room flooring materials	Tatami (Japanese straw floor covering)	755	(7.9)	1,716	(11.0)	3,452	(11.5)	3,238	(12.6)	< 0.001	0.04
	Carpet on tatami	636	(6.6)	1,347	(8.6)	2,781	(9.3)	2,422	(9.4)		
	Wooden flooring / tiles	3,760	(39.3)	5,302	(33.9)	10,417	(34.7)	9,224	(36.0)		
	Carpet on wooden flooring/tiles	4,249	(44.4)	6,964	(44.5)	12,866	(42.8)	10,311	(40.2)		
	Other	172	(1.8)	311	(2.0)	523	(1.7)	461	(1.8)		
Having a pet	No	7,529	(78.7)	11,917	(76.2)	23,136	(77.0)	19,921	(77.6)	< 0.001	0.02
	Yes	2,044	(21.4)	3,727	(23.8)	6,927	(23.0)	5,753	(22.4)		
Usage of air purifiers	Yes	5,017	(52.4)	7,685	(49.1)	15,215	(50.6)	13,235	(51.6)	< 0.001	0.02
	No	4,559	(47.6)	7,970	(50.9)	14,861	(49.4)	12,440	(48.5)		
Age of house/apartment building, y	<1	564	(5.9)	847	(5.4)	1,748	(5.8)	1,434	(5.6)	< 0.001	0.03
	1 – <3	1,176	(12.3)	1,811	(11.6)	3,403	(11.4)	2,748	(10.7)		
	3 – <5	981	(10.3)	1,549	(9.9)	2,871	(9.6)	2,300	(9.0)		
	5– <10	1,606	(16.9)	2,454	(15.7)	4,599	(15.4)	3,860	(15.1)		
	10 – <20	2,218	(23.3)	3,495	(22.4)	7,046	(23.5)	6,070	(23.7)		
	≥20	2,057	(21.6)	4,143	(26.6)	7,619	(25.4)	6,434	(25.2)		
	Unknown	926	(9.7)	1,298	(8.3)	2,684	(9.0)	2,729	(10.7)		
House renovation/interior finishing after getting pregnant	Yes	285	(3.0)	506	(3.2)	962	(3.2)	807	(3.2)	0.676	0.00
	No	9,264	(97.0)	15,089	(96.8)	29,006	(96.8)	24,782	(96.9)		
Number of years living in the current place of residence	<1	759	(8.1)	964	(6.3)	2,035	(7.0)	1,615	(6.5)	< 0.001	0.04
	1 – <3	4,527	(48.5)	6,653	(43.4)	12,641	(43.2)	10,251	(41.2)		
	3 – <5	1,987	(21.3)	3,431	(22.4)	6,560	(22.4)	5,862	(23.6)		
	5– <10	1,397	(15.0)	2,621	(17.1)	5,124	(17.5)	4,876	(19.6)		
	10 – <20	386	(4.1)	857	(5.6)	1,612	(5.5)	1,347	(5.4)		
	≥20	275	(3.0)	791	(5.2)	1,274	(4.4)	906	(3.6)		

Table S4. Characteristics of the participants with respect to the use of anti-mite bedding covers

Variable	Category	Using anti-mite cover for futon or bedding after getting pregnant					
		No		Yes			Cramer's
		N	(%)	N	(%)	*p*	*V*
Subtotal		74,757	(92.2)	6,349	(7.8)		
Age, y	<25	7,369	(9.9)	602	(9.5)	0.014	0.01
	25–<30	21,239	(28.4)	1,830	(28.8)		
	30–<35	26,536	(35.5)	2,351	(37.0)		
	≥35	19,606	(26.2)	1,565	(24.7)		
Body mass index, kg/m^2	<18.5	12,164	(16.3)	965	(15.2)	< 0.001	0.01
	18.5 – <25	55,060	(73.7)	4,662	(73.5)		
	≥25	7,493	(10.0)	719	(11.3)		
Parity	Primipara	31,611	(43.4)	2,639	(42.6)	0.207	0.00
	Multipara	41,261	(56.6)	3,563	(57.5)		
History of allergy	No	37,388	(50.2)	2,837	(44.9)	< 0.001	0.03
	Yes	37,067	(49.8)	3,481	(55.1)		
Number of hours spent outdoors, hour	<5	53,657	(71.9)	4,432	(69.9)	< 0.001	0.01
	5–12	18,808	(25.2)	1,674	(26.4)		
	≥13	2,216	(3.0)	233	(3.7)		
Smoking status	Never	43,828	(59.1)	3,566	(56.5)	< 0.001	0.01
	Former	27,406	(36.9)	2,471	(39.2)		
	Current	2,970	(4.0)	275	(4.4)		
Alcohol intake	Never	24,798	(33.4)	2,117	(33.6)	0.613	0.00
	Former	47,370	(63.8)	4,017	(63.8)		
	Current	2,051	(2.8)	161	(2.6)		
Number of hours spent outdoors	<1	13,861	(19.3)	1,044	(17.1)	< 0.001	0.02
	1–<2	34,675	(48.2)	2,836	(46.3)		
	2–<3	11,223	(15.6)	1,083	(17.7)		
	≥3	12,177	(16.9)	1,161	(19.0)		
Physical activity	No	16,793	(23.2)	1,248	(20.3)	< 0.001	0.02
	Yes	55,636	(76.8)	4,899	(79.7)		
Quintile of folic acid intake, μg	≤153	14,711	(19.7)	1,053	(16.6)	< 0.001	0.04
	154–203	15,110	(20.2)	1,175	(18.5)		
	204–257	15,147	(20.3)	1,253	(19.7)		
	258–337	15,100	(20.2)	1,325	(20.9)		
	≥338	14,682	(19.6)	1,542	(24.3)		
Marital status	Married	70,974	(95.7)	6,050	(96.1)	0.102	0.01
	Single	2,610	(3.5)	193	(3.1)		
	Divorced or widowed	572	(0.8)	56	(0.9)		
Highest education level, y	≤12	26,166	(35.1)	2,185	(34.6)	0.008	0.01
	>12 – <16	31,618	(42.4)	2,795	(44.3)		
	≥16	16,733	(22.5)	1,334	(21.1)		
Employed	No	33,759	(45.5)	2,899	(46.0)	0.422	0.00
	Yes	40,475	(54.5)	3,403	(54.0)		
Annual household income, million yen	<4	27,732	(39.7)	2,171	(36.8)	< 0.001	0.02
	4 – <6	23,218	(33.2)	2,005	(34.0)		
	≥6	18,900	(27.1)	1,728	(29.3)		
Type of residence	Wooden detached house	30,643	(41.2)	2,637	(41.7)	0.024	0.01
	Steel-frame detached house	4,663	(6.3)	445	(7.0)		
	Wooden multiple dwelling house/apartment	9,206	(12.4)	738	(11.7)		
	Steel-frame multiple dwelling house/apartment	29,179	(39.2)	2,426	(38.4)		
	Other	700	(0.9)	71	(1.1)		
Number of rooms in the house/apartment	≤2	14,082	(18.9)	1,130	(17.9)	< 0.001	0.02
	3	24,150	(32.5)	2,001	(31.7)		
	4	14,145	(19.0)	1,244	(19.7)		
	5	11,216	(15.1)	877	(13.9)		
	≥6	10,826	(14.6)	1,057	(16.8)		
Living room flooring materials	Tatami (Japanese straw floor covering)	8,500	(11.4)	661	(10.4)	< 0.001	0.02
	Carpet on tatami	6,642	(8.9)	544	(8.6)		
	Wooden flooring / tiles	26,260	(35.2)	2,443	(38.5)		
	Carpet on wooden flooring/tiles	31,823	(42.7)	2,567	(40.5)		
	Other	1,342	(1.8)	125	(2.0)		
Having a pet	No	57,642	(77.3)	4,861	(76.7)	0.323	0.00
	Yes	16,975	(22.8)	1,476	(23.3)		
Usage of air purifiers	Yes	37,132	(49.8)	4,020	(63.4)	< 0.001	0.07
	No	37,512	(50.3)	2,318	(36.6)		
Age of house/apartment building, y	<1	4,112	(5.5)	481	(7.6)	< 0.001	0.03
	1 – <3	8,363	(11.3)	775	(12.3)		
	3 – <5	7,112	(9.6)	589	(9.3)		
	5– <10	11,572	(15.6)	947	(15.0)		
	10 – <20	17,342	(23.3)	1,487	(23.6)		
	≥20	18,736	(25.2)	1,517	(24.0)		
	Unknown	7,118	(9.6)	519	(8.2)		
House renovation/interior finishing after getting pregnant	Yes	2,256	(3.0)	304	(4.8)	< 0.001	0.03
	No	72,134	(97.0)	6,007	(95.2)		
Number of years living in the current place of residence	<1	4,864	(6.7)	509	(8.3)	< 0.001	0.03
	1 – <3	31,283	(43.1)	2,789	(45.6)		
	3 – <5	16,531	(22.8)	1,309	(21.4)		
	5– <10	13,043	(18.0)	975	(15.9)		
	10 – <20	3,905	(5.4)	297	(4.9)		
	≥20	3,010	(4.1)	236	(3.9)		

Table S5. Prevalence, crude and adjusted odds ratios (ORs), and 95% confidence intervals (CIs) for the cases of developmental delay in each area assessed using the ASQ-3 according to the exposure variables

	6 months						12 months					
	Prevalence, %	Crude		Adjusted		*p*-value for trend	Prevalence, %	Crude		Adjusted		*p*-value for trend
		OR	[95% CI]	OR	[95% CI]			OR	[95% CI]	OR	[95% CI]	
Cleaning the living room floor with a vacuum cleaner												
Communication												
≤ 1–2 times a month	2.22	1.00	—	1.00	—	**0.017**	4.68	1.00	—	1.00	—	**< 0.001**
Once a week	1.95	0.88	[0.73, 1.06]	0.83	[0.69, 1.00]		3.66	**0.77**	**[0.67, 0.89]**	**0.81**	**[0.71, 0.94]**	
A few times a week	1.84	**0.83**	**[0.69, 0.99]**	**0.76**	**[0.63, 0.92]**		2.97	**0.62**	**[0.54, 0.71]**	**0.75**	**[0.65, 0.87]**	
Everyday	1.96	0.88	[0.72, 1.09]	**0.76**	**[0.61, 0.95]**		2.49	**0.52**	**[0.44, 0.61]**	**0.68**	**[0.57, 0.81]**	
Gross motor												
≤ 1–2 times a month	2.06	1.00	—	1.00	—	**0.016**	6.16	1.00	—	1.00	—	**< 0.001**
Once a week	1.93	0.94	[0.76, 1.15]	0.92	[0.75, 1.14]		5.92	0.96	[0.85, 1.08]	0.93	[0.82, 1.04]	
A few times a week	1.69	**0.81**	**[0.67, 0.99]**	**0.77**	**[0.63, 0.94]**		5.19	**0.83**	**[0.74, 0.93]**	**0.86**	**[0.76, 0.97]**	
Everyday	1.92	0.93	[0.74, 1.17]	0.82	[0.65, 1.04]		4.81	**0.77**	**[0.68, 0.88]**	**0.80**	**[0.70, 0.92]**	
Fine motor												
≤ 1–2 times a month	2.99	1.00	—	1.00	—	**< 0.001**	6.79	1.00	—	1.00	—	**< 0.001**
Once a week	2.83	0.94	[0.80, 1.11]	0.89	[0.75, 1.04]		6.13	0.90	[0.80, 1.00]	0.90	[0.81, 1.01]	
A few times a week	2.50	**0.83**	**[0.71, 0.97]**	**0.76**	**[0.65, 0.90]**		5.21	**0.75**	**[0.68, 0.84]**	**0.76**	**[0.68, 0.86]**	
Everyday	2.51	**0.83**	**[0.70, 1.00]**	**0.71**	**[0.58, 0.86]**		4.66	**0.67**	**[0.59, 0.76]**	**0.66**	**[0.58, 0.76]**	
Problem solving												
≤ 1–2 times a month	4.86	1.00	—	1.00	—	**< 0.001**	6.51	1.00	—	1.00	—	**< 0.001**
Once a week	3.80	**0.77**	**[0.68, 0.88]**	**0.77**	**[0.67, 0.88]**		5.77	**0.88**	**[0.79, 0.99]**	0.96	[0.85, 1.07]	
A few times a week	3.60	**0.73**	**[0.65, 0.83]**	**0.71**	**[0.62, 0.82]**		4.71	**0.71**	**[0.63, 0.80]**	**0.85**	**[0.76, 0.96]**	
Everyday	3.77	**0.77**	**[0.66, 0.89]**	**0.70**	**[0.60, 0.82]**		3.92	**0.59**	**[0.51, 0.67]**	**0.74**	**[0.64, 0.85]**	
Personal-social												
≤ 1–2 times a month	1.54	1.00	—	1.00	—	**0.018**	3.34	1.00	—	1.00	—	**0.009**
Once a week	1.44	0.93	[0.74, 1.17]	0.89	[0.70, 1.12]		3.03	0.91	[0.77, 1.06]	0.91	[0.78, 1.07]	
A few times a week	1.34	0.86	[0.69, 1.08]	**0.75**	**[0.59, 0.95]**		2.89	0.86	[0.74, 1.01]	0.86	[0.73, 1.01]	
Everyday	1.56	1.01	[0.79, 1.29]	0.77	[0.60, 1.00]		2.82	0.84	[0.70, 1.00]	**0.79**	**[0.65, 0.95]**	
Cleaning the futon with a vacuum cleaner												
Communication												
Almost never or never	2.13	1.00	—	1.00	—	**< 0.001**	3.83	1.00	—	1.00	—	**< 0.001**
A few times a year	1.98	0.93	[0.80, 1.09]	0.94	[0.80, 1.10]		3.07	**0.80**	**[0.70, 0.90]**	**0.81**	**[0.72, 0.92]**	
1–2 times a month	1.59	**0.74**	**[0.63, 0.86]**	**0.78**	**[0.67, 0.92]**		2.35	**0.60**	**[0.53, 0.68]**	**0.68**	**[0.60, 0.78]**	
≥ Once a week	1.46	**0.68**	**[0.57, 0.81]**	**0.77**	**[0.64, 0.92]**		2.13	**0.55**	**[0.47, 0.63]**	**0.71**	**[0.61, 0.82]**	
Gross motor												

Almost never or never	2.00	1.00	—	1.00	—	**0.017**	5.79	1.00	—	1.00	—	**0.010**
A few times a year	1.61	**0.80**	**[0.67, 0.95]**	**0.79**	**[0.66, 0.95]**		5.44	0.94	[0.85, 1.03]	0.94	[0.85, 1.03]	
1–2 times a month	1.74	0.87	[0.74, 1.02]	0.93	[0.79, 1.08]		5.04	**0.86**	**[0.79, 0.94]**	0.94	[0.86, 1.03]	
≥ Once a week	1.48	**0.74**	**[0.62, 0.88]**	**0.81**	**[0.68, 0.97]**		4.47	**0.76**	**[0.69, 0.84]**	**0.88**	**[0.80, 0.98]**	
Fine motor												
Almost never or never	2.96	1.00	—	1.00	—	**< 0.001**	6.00	1.00	—	1.00	—	**< 0.001**
A few times a year	2.72	0.92	[0.80, 1.05]	0.89	[0.78, 1.02]		5.39	**0.89**	**[0.81, 0.98]**	**0.89**	**[0.80, 0.98]**	
1–2 times a month	1.88	**0.63**	**[0.55, 0.72]**	**0.69**	**[0.60, 0.79]**		4.95	**0.82**	**[0.75, 0.89]**	**0.89**	**[0.81, 0.97]**	
≥ Once a week	2.19	**0.73**	**[0.64, 0.85]**	0.88	[0.76, 1.02]		4.5	**0.74**	**[0.67, 0.81]**	**0.87**	**[0.79, 0.96]**	
Problem solving												
Almost never or never	4.20	1.00	—	1.00	—	**< 0.001**	5.84	1.00	—	1.00	—	**< 0.001**
A few times a year	3.82	0.91	[0.81, 1.01]	**0.89**	**[0.80, 1.00]**		4.73	**0.80**	**[0.72, 0.89]**	**0.81**	**[0.73, 0.89]**	
1–2 times a month	3.17	**0.75**	**[0.67, 0.83]**	**0.82**	**[0.73, 0.92]**		3.96	**0.67**	**[0.60, 0.73]**	**0.74**	**[0.67, 0.82]**	
≥ Once a week	2.90	**0.68**	**[0.60, 0.77]**	**0.79**	**[0.70, 0.90]**		3.48	**0.58**	**[0.52, 0.65]**	**0.70**	**[0.63, 0.79]**	
Personal-social												
Almost never or never	1.59	1.00	—	1.00	—	**0.001**	3.32	1.00	—	1.00	—	**< 0.001**
A few times a year	1.25	**0.78**	**[0.64, 0.96]**	**0.75**	**[0.61, 0.93]**		2.94	0.88	[0.77, 1.01]	**0.87**	**[0.76, 0.99]**	
1–2 times a month	1.21	**0.76**	**[0.63, 0.91]**	**0.78**	**[0.64, 0.93]**		2.38	**0.71**	**[0.63, 0.81]**	**0.76**	**[0.67, 0.86]**	
≥ Once a week	1.18	**0.74**	**[0.60, 0.90]**	**0.78**	**[0.63, 0.96]**		2.22	**0.66**	**[0.58, 0.76]**	**0.75**	**[0.65, 0.87]**	
Airing the futon												
Communication												
Almost never or never	2.16	1.00	—	1.00	—	0.304	4.26	1.00	—	1.00	—	**< 0.001**
A few times a year	1.87	0.86	[0.72, 1.04]	0.84	[0.70, 1.02]		3.55	**0.83**	**[0.72, 0.94]**	0.88	[0.76, 1.00]	
1–2 times a month	1.94	0.89	[0.76, 1.05]	0.91	[0.77, 1.07]		3.27	**0.76**	**[0.67, 0.86]**	**0.87**	**[0.77, 0.99]**	
≥ Once a week	1.86	0.86	[0.73, 1.02]	0.87	[0.73, 1.03]		2.66	**0.61**	**[0.54, 0.70]**	**0.77**	**[0.67, 0.88]**	
Gross motor												
Almost never or never	1.94	1.00	—	1.00	—	0.512	6.02	1.00	—	1.00	—	0.394
A few times a year	1.88	0.97	[0.80, 1.17]	0.94	[0.77, 1.14]		5.77	0.96	[0.86, 1.07]	0.97	[0.87, 1.09]	
1–2 times a month	1.79	0.92	[0.77, 1.09]	0.90	[0.76, 1.08]		5.31	**0.87**	**[0.79, 0.97]**	0.93	[0.84, 1.03]	
≥ Once a week	1.82	0.94	[0.78, 1.12]	0.94	[0.78, 1.13]		5.17	**0.85**	**[0.77, 0.94]**	0.96	[0.86, 1.07]	
Fine motor												
Almost never or never	3.03	1.00	—	1.00	—	**0.018**	6.00	1.00	—	1.00	—	0.051
A few times a year	2.90	0.96	[0.82, 1.12]	0.94	[0.81, 1.10]		6.13	1.02	[0.92, 1.14]	1.01	[0.91, 1.13]	
1–2 times a month	2.50	**0.82**	**[0.72, 0.95]**	**0.85**	**[0.74, 0.98]**		5.40	**0.89**	**[0.81, 0.99]**	0.93	[0.84, 1.03]	
≥ Once a week	2.51	**0.82**	**[0.71, 0.95]**	**0.85**	**[0.73, 0.99]**		5.16	**0.85**	**[0.77, 0.94]**	0.93	[0.83, 1.03]	
Problem solving												
Almost never or never	4.40	1.00	—	1.00	—	**0.001**	6.18	1.00	—	1.00	—	**0.002**
A few times a year	4.21	0.96	[0.84, 1.09]	0.93	[0.82, 1.07]		5.77	0.93	[0.83, 1.04]	0.95	[0.85, 1.06]	
1–2 times a month	3.53	**0.80**	**[0.71, 0.89]**	**0.81**	**[0.72, 0.91]**		4.81	**0.77**	**[0.69, 0.85]**	**0.85**	**[0.77, 0.94]**	

≥ Once a week	3.63	**0.82**	**[0.73, 0.92]**	**0.84**	**[0.74, 0.95]**		4.50	**0.72**	**[0.65, 0.80]**	**0.86**	**[0.77, 0.96]**	
Personal-social												
Almost never or never	1.41	1.00	—	1.00	—	0.493	3.52	1.00	—	1.00	—	**< 0.001**
A few times a year	1.47	1.05	[0.84, 1.30]	1.04	[0.83, 1.29]		3.25	0.92	[0.80, 1.06]	0.91	[0.79, 1.05]	
1–2 times a month	1.40	0.99	[0.80, 1.22]	0.98	[0.80, 1.21]		2.80	**0.79**	**[0.69, 0.90]**	**0.79**	**[0.69, 0.91]**	
≥ Once a week	1.43	1.01	[0.82, 1.24]	0.96	[0.77, 1.18]		2.76	**0.78**	**[0.68, 0.89]**	**0.76**	**[0.66, 0.88]**	
Using anti-mite cover for futon or bedding after getting pregnant												
Communication												
No	1.97	1.00	—	1.00	—	**0.037**	3.33	1.00	—	1.00	—	**0.005**
Yes	1.43	**0.72**	**[0.58, 0.91]**	**0.78**	**[0.62, 0.98]**		2.33	**0.69**	**[0.59, 0.82]**	**0.78**	**[0.65, 0.93]**	
Gross motor												
No	1.88	1.00	—	1.00	—	**0.010**	5.48	1.00	—	1.00	—	0.150
Yes	1.32	**0.70**	**[0.55, 0.87]**	**0.74**	**[0.59, 0.93]**		4.87	**0.88**	**[0.78, 0.99]**	0.91	[0.81, 1.03]	
Fine motor												
No	2.68	1.00	—	1.00	—	0.365	5.67	1.00	—	1.00	—	**< 0.001**
Yes	2.28	0.85	[0.71, 1.01]	0.92	[0.77, 1.10]		3.96	**0.69**	**[0.60, 0.78]**	**0.74**	**[0.65, 0.85]**	
Problem solving												
No	3.86	1.00	—	1.00	—	**0.020**	5.19	1.00	—	1.00	—	**< 0.001**
Yes	3.00	**0.77**	**[0.66, 0.90]**	**0.83**	**[0.71, 0.97]**		3.52	**0.67**	**[0.58, 0.77]**	**0.74**	**[0.64, 0.85]**	
Personal-social												
No	1.44	1.00	—	1.00	—	0.495	3.02	1.00	—	1.00	—	**0.009**
Yes	1.23	0.85	[0.66, 1.10]	0.92	[0.71, 1.18]		2.21	**0.72**	**[0.60, 0.87]**	**0.79**	**[0.66, 0.94]**	

OR, odds ratio; CI, confidence interval; ASQ-3, Ages and Stages Questionnaire, Third Edition.

Based on imputed data for the 81,106 infants enrolled in this study.

Boldface indicates statistical significance at the level of 5%.

P-values for trend were calculated for adjusted models.

Crude=crude model.

Adjusted = model adjusted for maternal age, pre-pregnancy body mass index, parity, history of allergy, psychological distress, smoking status, alcohol intake, number of hours spent outdoors, physical activity, folic acid intake, marital status, highest educational level, employment status, annual household income, type of residence, number of rooms in the house/apartment, living room flooring material, having a pet, usage of air purifiers, age of house/apartment building, house renovation/interior completion after becoming pregnant, and number of years living in the current place of residence.
